# To choose or not to choose

**DOI:** 10.7554/eLife.66755

**Published:** 2021-02-26

**Authors:** Supriya Srinivasan

**Affiliations:** Department of Neuroscience and Dorris Neuroscience Center, The Scripps Research InstituteLa JollaUnited States

**Keywords:** aging, nutrient choice, metabolism, tca cycle, serotonin, *D. melanogaster*

## Abstract

Making choices about food affects the metabolism and lifespan of fruit flies.

**Related research article** Lyu Y, Weaver KJ, Shaukat HA, Plumoff ML, Tjilos M, Promislow DE, Pletcher SD. 2021. Serotonin 2A receptor signaling coordinates central metabolic processes to modulate aging in response to nutrient choice. *eLife* **10**:e59399. doi: 10.7554/eLife.59399

Vegan, macrobiotic or Atkins – what constitutes a good diet remains a matter of continuous debate. From early studies in the 1950s investigating the health benefits of macronutrients (that is, the relative ratios of carbohydrates, proteins and fats) to current research showing that caloric restriction increases lifespan, scientists and lay people alike remain fascinated by the relationship between diet and longevity (Keys et al., 1957; McCay et al., 1989).

The typical way of addressing such questions in a laboratory setting would be to compare groups given diets with different macronutrient compositions and measure their lifespans. But in real life, food is neither presented nor consumed that way. First, foods vary in their composition of macronutrients (fruits and vegetables contain mostly carbohydrates, meat is mostly protein, and seeds and nuts contain mostly fats). Second, all creatures tend to have innate preferences towards certain foods. Taking these discrepancies into account, would we see a connection between macronutrients and longevity in a more naturalistic, choice-based food environment?

Now, in eLife, Scott Pletcher of the University of Michigan and colleagues – including Yang Lyu as first author and co-workers at Michigan and the University of Washington – report that both our diet and our food choices can influence metabolism and lifespan (Lyu et al., 2021). The researchers set out to address this topic in a widely used model system, the fruit fly *Drosophila melanogaster.* In the first set of experiments, one group of wild-type fruit flies spent their lives on a diet consisting of equal amounts of sugar and protein (fixed diet). The second group received the same amount and ratio of sugar and protein, but they could choose between the two foods (choice diet). Then, the amount of food consumed and lifespans were measured and compared across both groups. Alas – and perhaps unsurprisingly – flies given the choice between sugar and protein consumed far more sugar and lived less long than those given no choice in the matter (Figure 1).

**Figure 1.**
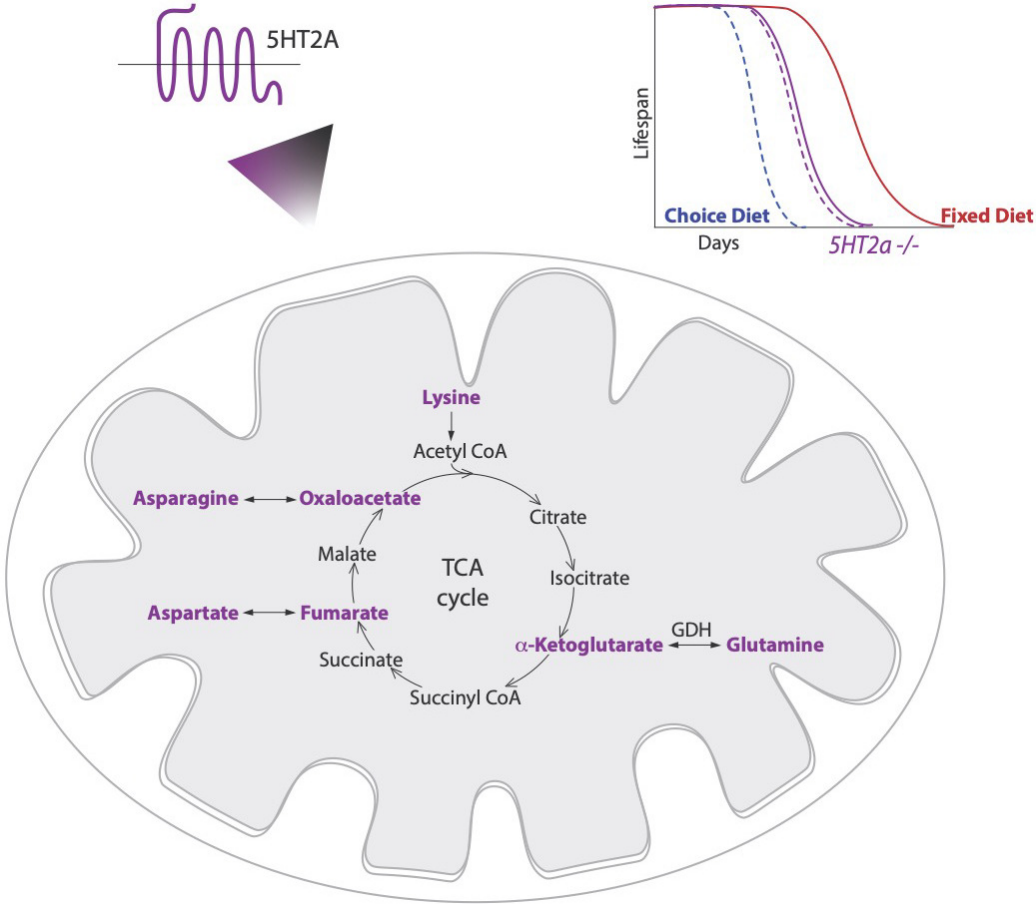
Diet choice influences metabolic pathways and lifespan. Lyu et al. found that fruit flies given a choice between sugar and protein preferentially consume sugar and live a shorter life (dashed blue line; top right) than those given a fixed diet of equal proportions of sugar and protein (solid red line). A serotonin receptor called 5HT2A controls longevity in flies on a choice diet but has little influence on the lifespan of mutant flies that lacked 5HT2A (dashed and solid purple lines). Lyu et al. also measured the levels of various metabolites involved in the conversion of food into energy in flies raised on both diets, and with or without the 5HT2A receptor. This revealed that certain intermediates (highlighted in purple) of the tricaboxylic acid cycle (TCA), which can interconvert to amino acids, were upregulated on the sugar-rich choice diet in a 5HT2A-dependent manner. This indicates a functional link between serotonin signaling, metabolic rearrangements and longevity.

However, the reduced longevity of the ‘sweet-toothed’ flies could not be attributed solely to sugar-induced toxicity. Even flies given up to three times as much sugar as protein in the fixed-diet group had an intermediate lifespan. Additionally, other measures, such as intestinal permeability and locomotion, were unaltered by the choice diet, at least in young flies. Not even a major messenger molecule in the insulin signaling pathway (dFOXO), a key culprit in diet-induced longevity, was greatly affected by a choice-driven diet (Kenyon et al., 1993). Instead, it appeared that being presented with the choice itself led to increased sugar consumption and reduced longevity.

Based on a hunch from their previous work (Ro et al., 2016), Lyu et al. were able to show that a serotonin receptor called 5HT2A was responsible for the choice-induced reduction in lifespan. However, when 5HT2A was removed, flies on a choice diet no longer had shortened lifespans, even though they consumed just as much sugar as the wild-type flies. Indeed, previous research has shown that serotonin signaling in neurons can alter the metabolic state in the gut and also control longevity (Littlejohn et al., 2020; Palamiuc et al., 2017; Srinivasan et al., 2008).

Lyu et al. suspected that variations in internal nutrients could be behind the observed changes in lifespan. They compared a large number of metabolites relevant for converting food to energy in flies raised on both diets, and with or without 5HT2A. Over 80% of these metabolites did not change between any of the groups – a result that is both plausible and comforting, indicating that internal homeostasis is preserved in the contrasting diets. However, in flies raised on a choice-diet, four amino acids (lysine, glutamine, asparagine and aspartate) increased in a 5HT2A-dependent manner. Interestingly, these metabolites also have the capacity to interconvert between different energy substrates (Figure 1).

Moreover, the precursors of these amino acids (including α-ketoglutarate, which is a precursor of glutamine) also increased in the choice diet. These precursors are also intermediates in the tricaboxylic acid cycle (also known as the Krebs or citric acid cycle), which converts various nutrient sources into molecules called ATP and NADH, which store the chemical energy that cells rely on to carry out a wide range of cellular functions. A second critical function of this cycle is to supply non-essential amino acids if they are not present in the diet (Salway, 1999).

Lyu et al. further investigated the role of an enzyme called glutamate dehydrogenase, which helps the cell to process sugars by interconverting α-ketoglutarate to glutamine via glutamate and back, depending on the energetic state of the cell. Flies without glutamate dehydrogenase had a normal lifespan on the choice diet, suggesting that some aspect of the flux between α-ketoglutarate and glutamine is deleterious to longevity. Whether these metabolites are responsible for the decrease in longevity or innocent bystanders is still unclear. Nevertheless, Lyu et al. provide new evidence that food influences the chemistry of the brain, and that the signals from the brain in turn can affect food choices, metabolism and aging.

Despite these intriguing findings, many questions remain. First, how and where does neuronal 5HT2A signaling alter the tricaboxylic acid cycle and the flux of amino acids? The mammalian ortholog of the 5HT2A receptor is a target of psychoactive drugs, including LSD and psilocybin, and is also known to be downregulated by long-term antidepressant use (Gawliński et al., 2019). Lyu et al. further discovered that another vital neuromodulator (called kyneurenine) – which links energy production, immune and neuroendocrine functions – is highly upregulated in flies on the choice diet (Oxenkrug, 2010). Could there be a causal link between these neural signaling molecules, diet and longevity? Moreover, in what way, and in what proportions, would including fats into such studies modulate longevity across different species? While these results suggest that our food choices alone are sufficient to influence metabolic reactions, more research is needed before we can tackle that age-old question: what should I eat to live long and prosper?

## References

[bib1] Gawliński D, Smaga I, Zaniewska M, Gawlińska K, Faron-Górecka A, Filip M (2019). Adaptive mechanisms following antidepressant drugs: focus on serotonin 5-HT2A receptors. Pharmacological Reports.

[bib2] Kenyon C, Chang J, Gensch E, Rudner A, Tabtiang R (1993). A *C. elegans* mutant that lives twice as long as wild type. Nature.

[bib3] Keys A, Anderson J, Grande F (1957). Prediction of serum-cholesterol responses of man to changes in fats in the diet. The Lancet.

[bib4] Littlejohn NK, Seban N, Liu CC, Srinivasan S (2020). A feedback loop governs the relationship between lipid metabolism and longevity. eLife.

[bib5] Lyu Y, Weaver KJ, Shaukat HA, Plumoff ML, Tjilos M, Promislow DE, Pletcher SD (2021). Serotonin 2A receptor signaling coordinates central metabolic processes to modulate aging in response to nutrient choice. eLife.

[bib6] McCay CM, Crowell MF, Maynard LA (1989). The effect of retarded growth upon the length of life span and upon the ultimate body size. 1935. Nutrition.

[bib7] Oxenkrug GF (2010). Metabolic syndrome, age-associated neuroendocrine disorders, and dysregulation of tryptophan-kynurenine metabolism. Annals of the New York Academy of Sciences.

[bib8] Palamiuc L, Noble T, Witham E, Ratanpal H, Vaughan M, Srinivasan S (2017). A tachykinin-like neuroendocrine signalling axis couples central serotonin action and nutrient sensing with peripheral lipid metabolism. Nature Communications.

[bib9] Ro J, Pak G, Malec PA, Lyu Y, Allison DB, Kennedy RT, Pletcher SD (2016). Serotonin signaling mediates protein valuation and aging. eLife.

[bib10] Salway JG (1999). Metabolism at a Glance.

[bib11] Srinivasan S, Sadegh L, Elle IC, Christensen AG, Faergeman NJ, Ashrafi K (2008). Serotonin regulates *C. elegans* fat and feeding through independent molecular mechanisms. Cell Metabolism.

